# The Nervous System and Its Diseases: A Practical Treatise on Neurology, for the Use of Physicians and Students

**Published:** 1898-06

**Authors:** 


THE NERVOUS SYSTEM AND ITS DISEASES: A Practical Treatise on Neurology, for the Use of Physicians and Students. By CHARLES K. MILLS, M. D., Professor of Mental Diseases and of Medical Jurisprudence in the University of Pennsylvania; Clinical Professor of Neurology in the Woman's Medical College of Pennsylvania; Professor of Diseases of the Nervous System in the Philadelphia Polyclinic; Neurologist to the Philadelphia Hospital, etc. Royal octavo, pp. xxx.—1,056. With 459 illustrations. Philadelphia: J. B. Lippincott Company. 1898.

The author has planned a most elaborate and complete presentation of the nervous system and its diseases. The present volume is not only abreast of the times, but it actually leads in the advanced discussion of the recent additions to the anatomy and pathology of the nervous system.

The student who uses this work as his first on nervous diseases will have the advantage of not being required to unlearn the old nomenclature. Throughout the work the nomenclature and terminology advocated by Prof. Wilder, of Cornell, has been used. The adoption of the reforms by the author is of great significance and will do much toward making their use general in future text-books.

As Mills says, "The reforms advocated by this distinguished anatomist, especially the introduction of mononyms, are deserving of general adoption."

The first chapter of 124 pages is devoted to a general sketch of the nervous system, its tissues, development, anatomy, physiology, nomenclature and chemistry.

In this section the author dissents from the view that the sensory tract terminates in the 'cortical motor zone. He believes that the cortical motor and sensory areas are practically separate.

His discussion of the architecture and general physiology of the nervous system is very clear and must be thoroughly understood in order to follow him in his discussion of localisation.

He considers in Chapter II. general pathology, etiology, symptomatology—methods of investigation, electricity and general therapeutics. Under general therapeutics we find careful consideration of balneotherapy and the diseases in which various forms of baths are useful—massage, movement treatment, vibratory therapeutics, Weir Mitchell's rest cure with Schedall's suspension, hypnotism and local remedies. The uses of animal extracts of cerebrin, he says, "the subject may be considered unsettled with the weight of authority against it." Of medullin pancreatin, gastrin cardin, he says, "some of the results obtained are scarcely worthy of mention." Of testicular therapy he accepts the conclusions of Venturi and Frondo, that testicular remedies have given negative or, at best, only transitory results.

The chapter is made unusually valuable by the very full discussion of the uses of the more potent drugs and a statement of the author's experience with them. In the third chapter is presented diseases of the membranes, sinuses and veins of the brain, brain malformations and aberrations.

In the fourth chapter he considers encephalic histology and physiology in their relation to focal diseases. Here we find the latest and best study of cortical localisation. He does not force his own views, but gives fairly the opinions of those who differ from him. He is not so disputatious as Ferrier, but presents his case with just as much strength. This is a quality which carries great weight and adds pleasure to the reader's study.

In the fifth chapter is a study of the encephalic vessels and their diseases.

In the sixth we find a full discussion of aphasia and notice with interest the large cortical area which is now allotted to speech.

In the remaining chapters is a systematic consideration of the affections of the special senses and cranial nerves. This includes a special section on the cochlear and vestibular nerves and their diseases.

The entire volume is most satisfactory and bears the stamp of a master. The profession will be anxious for the second volume, which will consider the spinal cord and its diseases and the functional and diseases of peripheral nerves. The work of the publishers in illustration and letter-press is of the best. J. W. P.

